# Identifying barriers and enablers to participation in infection surveillance in Australian residential aged care facilities

**DOI:** 10.1186/s12889-023-16891-2

**Published:** 2023-11-04

**Authors:** Eliza Watson, Leslie Dowson, David Dunt, Karin Thursky, Leon J. Worth, Janet K. Sluggett, Amanda Appathurai, Noleen Bennett, for the National Infection Surveillance Program in Aged Care (NISPAC) Advisory Group

**Affiliations:** 1 Victorian Healthcare Associated Infection Surveillance System (VICNISS) Coordinating Centre, Melbourne, VIC 3000 Australia; 2 National Centre for Antimicrobial Stewardship, Department of Infectious Diseases, The University of Melbourne, Parkville, VIC 3010 Australia; 3 Professor Emeritus, The University of Melbourne, Parkville, VIC 3010 Australia; 4 Sir Peter MacCallum Department of Oncology, The University of Melbourne, Parkville, VIC 3010 Australia; 5 The Royal Melbourne Hospital Guidance Group, Melbourne, VIC 3000 Australia; 6 Department of Medicine, The University of Melbourne, Parkville, VIC 3010 Australia; 7 University of South Australia, UniSA Allied Health and Human Performance, Adelaide, SA 5001 Australia; 8 Registry of Senior Australians (ROSA), Healthy Ageing Research Consortium, South Australian Health and Medical Research Institute, Adelaide, SA 5001 Australia; 9 Department of Nursing, Melbourne School of Health Sciences, The University of Melbourne, Parkville, VIC 3010 Australia

**Keywords:** Nursing home, Cross infection, Surveillance and Qualitative research

## Abstract

**Background:**

Infection surveillance is a vital part of infection prevention and control activities for the aged care sector. In Australia there are two currently available infection and antimicrobial use surveillance programs for residential aged care facilities. These programs are not mandated nor available to all facilities. Development of a new surveillance program will provide standardised surveillance for all facilities in Australia.

**Methods:**

This study aimed to assess barriers and enablers to participation in the two existing infection and antimicrobial use surveillance programs, to improve development and implementation of a new program. A mixed-methods study was performed. Aged Care staff involved in infection surveillance were invited to participate in focus groups and complete an online survey comprising 17 items. Interviews were transcribed and analysed using the COM-B framework.

**Results:**

Twenty-nine staff took part in the focus groups and two hundred took part in the survey. Barriers to participating in aged care infection surveillance programs were the time needed to collect and enter data, competing priority tasks, limited understanding of surveillance from some staff, difficulty engaging clinicians, and staff fatigue after the COVID-19 pandemic. Factors that enabled participation were previous experience with surveillance, and sharing responsibilities, educational materials and using data for benchmarking and to improve practice.

**Conclusion:**

Streamlined and simple data entry methods will reduce the burden of surveillance on staff. Education materials will be vital for the implementation of a new surveillance program. These materials must be tailored to different aged care workers, specific to the aged care context and provide guidance on how to use surveillance results to improve practice.

**Supplementary Information:**

The online version contains supplementary material available at 10.1186/s12889-023-16891-2.

## Background

Infection prevention and control (IPC) activities are essential for the safety of residents, carers and staff in residential aged care facilities (RACFs) [1]; these facilities provide access to personal care, nursing and health services to older adults. Residents in RACFs are particularly vulnerable to infections [2, 3]. One cohort study found infections accounted for one quarter of all overnight hospitalisations from South Australian RACFs [4].

The Australian Aged Care Quality Standards outline how a RACF might demonstrate the minimization of infection-related risks; this includes the use of ‘data to monitor infections and resolution rates and the effectiveness of an IPC program’ [5]. Monitoring and reporting of infections in healthcare and RACF settings has been shown to reduce risks for acquisition and burden of infection, through support for quality improvement activities [1, 6, 7]. Use of standardised surveillance methods improves validity of data and allows RACFs to benchmark and evaluate internal performance over time [1, 8, 9].

In Australia, there are two main surveillance programs configured for the monitoring of infection and antimicrobial use in RACFs – the Aged Care National Antimicrobial Prescribing Survey (NAPS) and the Victorian Healthcare Associated Infection Surveillance System (VICNISS) Coordinating Centre Aged Care Infection Indicator Program (ACIIP). Facilities which are publicly managed in the state of Victoria are required to participate in the VICNISS ACIIP. Aged Care NAPS is available to all RACFs nationally, but is not mandated [6, 10, 11]. Mandating participation in quality improvement activities in the aged care sector can motivate staff and lead to improved quality of care [9, 12].

The benefits of a national, standardised and mandated surveillance program are well recognised in Australia and internationally [13, 14]. Development of a National Infection Surveillance Program for Aged Care (NISPAC) for Australian RACFs is now underway. This program aims to deliver a standardised surveillance system, leveraging from the two currently available surveillance programs, in addition to newly developed surveillance modules (e.g. respiratory infection surveillance, including COVID-19). The current study will contribute towards the development of NISPAC through an evaluation of the merits and limitations of the two existing surveillance programs in Australia. This evaluation will ensure that NISPAC is developed and implemented in accordance with current resources, emerging risks and stakeholder needs, and is therefore implemented in a sustainable manner.

To date, no in-depth qualitative evaluation of either program has been performed. There is also limited international data on user attitudes towards similar programs [15]. This study aimed to assess barriers and enablers to participation in infection surveillance in Australian RACFs, to understand whether existing programs are acceptable to staff and to identify potential improvements for future program development.

## Method

This was a mixed methods study using a survey and focus groups in order to capture the views of healthcare staff employed in Australian RACFs. Michie et al.’s Behaviour Change Wheel (BCW) [16] was used as the analysis framework. The BCW outlines three key domains of behaviour – capability, opportunity and motivation, also referred to as COM-B.

This study received ethical approval from the Royal Melbourne Hospital Human Research Ethics Committee (HREC) (HREC/82249/MH-2022).

### Setting

Australia’s aged care system is managed both publicly through government-owned facilities, and privately through for-profit and not-for-profit facilities. There are approximately 805 approved aged care service providers in Australia, managing more than 2,600 RACFs [17]. Aged Care NAPS officially commenced in 2016 and is optional for most of these facilities; 568 RACFs participated nationally in 2019 [18]. Aged Care NAPS is an annual survey that allows users to conduct surveillance of antimicrobial use and infections (urinary tract, respiratory tract, skin and soft tissue, eye and ear) in their facilities.

The 179 publicly-operated RACFs in Victoria are required to participate in both Aged Care NAPS and the VICNISS ACIIP [11, 19]. The VICNISS ACIIP, commenced in 2017, allows users to conduct continuous surveillance of significant organism infections (Methicillin Resistance *Staphylococcus aureus,* Vancomycin Resistance enterococci, *Clostridioides difficile*), and point prevalence surveillance of staff influenza vaccination and resident influenza, herpes zoster and pneumococcal vaccination status.

As of October 2020, Australian RACFs have been required to employ a clinical lead with specific training in IPC (IPC leads) [20, 21]. IPC Leads, IPC Consultants (employed across multiple facilities to assist with IPC activities), Quality Managers and pharmacists are the workforce involved in infection surveillance in RACFs. Doctors are based offsite from RACFs, and those who visit residents are mostly primary care physicians (general practitioners). Personal Care Assistants (PCAs) are also employed by RACFs to assist with the care of residents, however they do not have the required qualifications to undertake surveillance.

### Participants

Purposive sampling was used to recruit participants who were currently employed by a RACF and who held registration with Aged Care NAPS and ACIIP. These staff included IPC Leads, IPC Consultant, Quality Managers and pharmacists. Email invitations were sent to mailing lists comprising 1852 registered users, noting it was unknown how many were still employed at their registered RACF(s). These mailing lists are hosted internally by the two programs, who granted permission for this research to take place in accordance with maintenance of privacy under the ethics agreement.; Invitations for the survey and focus groups were sent separately, with the survey invitation sent several months prior to the focus group invitations, due to delays in ethics approval. Survey participants were asked to complete an electronic consent form before gaining access to the survey. Those interested in participating in the focus groups completed an electronic consent form, eligibility form and provided preferences of availability for the focus groups, they were then contacted by a researcher and allocated to their preferred time or were placed on a waitlist if there were more participants than needed. Each focus group aimed to have 5–7 participants, approximately 9 were allocated to each account for potential fall out of participants, which was expected due to the busy schedules of RACF staff.

### Focus groups

Five focus groups were hosted in September 2022 online (Zoom® Video Communications Inc., San Jose, California, USA). One researcher (EW) facilitated the discussions using a semi-structured focus group guide (provided in Additional file 1), a second researcher (AA or NB) observed and wrote field notes during the focus groups. The focus group interview guide was developed based on the Capability, Opportunity and Motivation for Behaviour (COM-B) framework, and refined based on the results of the survey [16]. All focus groups were audio recorded and transcribed using the Zoom® automatic transcription function, the transcripts were then corrected by two of the researchers (EW and AA).

Five groups were arranged, with capacity for more if data saturation was not reached.

### Survey

Survey questions were primarily based on the Centers’ for Disease Control Updated Guidelines for Evaluating Public Health Surveillance Systems [United States of America (USA)] [22]. Questions were multiple choice, with some optional open-ended questions. The survey comprised of questions regarding infection surveillance resources at the participants’ RACFs and was divided into several sections. Results from 17 items from the following survey sections will be presented in this paper: Demographics, Human Resources (Infection Prevention and Control), Pathology, Information technology, Surveillance and Education. Participants were also questioned specifically about the ACIIP and Aged Care NAPS program.

Surveys were conducted on-line using REDcap electronic data capture tools hosted at Melbourne Health [23, 24]. The survey was open for a four-week period (May 2022), two reminder emails were sent during this period.

### Data analysis

Focus group transcripts were uploaded to NVivo®, version 12 (QSR International Pty. Ltd., Burlington, Massachusetts, USA) [22]. Transcripts were analysed thematically using a process of open, axial coding. Deductive coding was used to identify domains from COM-B, with concurrent inductive coding used to identify sub-themes categorised to each of the COM-B domains [16]. Coding of all transcripts was undertaken by one researcher (EW), with 20% independently coded by a second researcher (LD). Both researchers met to discuss coding and discrepancies.

Descriptive analysis of the survey data was performed using STATA®/SE 14.2 (StataCorp LLC., College Station, Texas, USA) to determine frequencies of responses [25].

The focus group and survey data were analysed separately. Triangulation was used to determine where results converged, were complimentary or contradictory [26]. Survey results were mapped to the themes identified from the qualitative data. Mapping of survey results enhanced the identified themes by providing more detail or contrasting to the focus group results.

## Results

### Participants

Two hundred RACF staff participated in the survey. Of these, 123 participants answered all questions. Most of the participants were IPC Leads (Table 1), largely employed by public (*n* = 93, 47.2%) and not-for-profit (*n* = 83, 42.1%) RACFs. Participants were mostly located in the state of Victoria (*n* = 84, 42.2%) followed by New South Wales (*n* = 34, 17.1%) with comparable representation of metropolitan (*n* = 61, 31.1%), regional (*n* = 74, 37.8%) and rural (*n* = 61, 31.3%) facilities.

**Table 1:** Positions of survey participants

Position	n (%)
IPC Lead	64 (32)
IPC Consultant	46 (23)
Nurse Unit or Site Manager	31 (15.5)
Pharmacist	17 (8.5)
Executive Manager	6 (3)
Other	36 (18)
Total	200

*IPC Lead* Infection Prevention and Control Lead, *IPC Consultant* Infection Prevention and Control Consultant

There were 29 participants across the five focus groups. Participants with different positions and from different facility types were spread across the focus groups, with IPC Leads (*n* = 13, 44.8%) and IPC Consultants (*n* = 9, 31%) being the most common roles. Participants were largely from RACFs in the state of Victoria (*n* = 20, 69%), and from not-for-profit (*n* = 13, 44.8%) or public (*n* = 10, 34.5%) facilities. Three participants (10.4%) were within 5 years of graduating from their qualification, while most of the participants were more than 20 years post-graduation (*n* = 16, 55.2%).

### Survey and focus group results

The survey results offered an insight into the operations of RACFs and their capacity for surveillance, including an initial understanding of some key barriers for staff participation in surveillance. Table 2) The focus groups had an average length of 44 min. Common themes emerged by the third focus group, and data saturation was evident following the fifth focus group. There was a high level of concordance between EW and LD’s coding and identification of major themes.

**Table 2:** Barriers to participation in an Infection and antimicrobial use surveillance program identified from the survey

Barriers	n (% of cases)^a^
Considerable time commitment	87 (69.6)
Competing priority tasks	60 (48)
Lack of a well-defined targeted surveillance plan	43 (34.4)
Lack of expertise in surveillance	37 (29.6)
Lack of targeted education	37 (29.6)
Limited onsite personnel	33 (26.4)
Limited access to Infectious Diseases expertise	30 (24)
There are no barriers to participation	23 (18.4)
Insufficient management support	21 (16.8)
Limited access to pathology reports	19 (15.2)
Limited immediate access to IPC support	18 (14.4)
Unsure where to start	18 (14.4)
Data not available	15 (12)
Other	7 (5.6)
Limited computer access	6 (4.8)
Lack of IT support	4 (3.2)
Total cases	125

^a^*Participants were able to select more than one option. The results are presented as a percentage of cases*

Themes are described below and are identified as barriers and enablers to participation in infection and antimicrobial use surveillance in RACFs, within each of the COM-B domains.

Complete survey results are provided in Additional File 2 and key quotes for each theme can be found in Table 3.

**Table 3:** Major themes and quotes from the focus groups

COM-B domain	Barrier or enabler	Theme	Quotes
Capability	Barrier	Infection surveillance not widely understood by RACF staff	*“they [staff] can talk that they know what McGeer [criteria] is, but I think only the ones that use the NAPS really have an understanding of how to apply it properly”* (Director of Quality and Governance, FG5) *“surveillance and understanding the McGeer [criteria] is just not a concept that is well understood in aged care. And I have got experience with this in trying to establish surveillance systems in my aged care Facilities, and some of them are not even aware of McGeer.”* (IPC Consultant 3, FG2)
	Enabler	Previous experience with infection surveillance improves understanding	*“My understanding of surveillance is very good.”* (IPC Consultant 3, FG2) *“So from my perspective Infections surveillance is looking at what type of infections have occurred in your facility, how they’ve been identified, also how they've been treated according to best practice or best guidelines.”* (Pharmacist 4, FG4) *“I definitely find that staff who've completed the survey before have a better experience than those who haven't, so practice makes perfect I think”* (Pharmacist 3, FG3) *“I think this will be our six year we've done it, and we get better at it every year, I have to say, and probably because our infection prevention component of our team gets better each year. … I think the useful thing that we are getting better at is probably the preparing for it, and so making sure that everybody is really clear exactly when we are going to be auditing. … Over the years, I guess we've got better at finding results, finding indications.”* (Quality Manager 1, FG1)
Opportunity	Barrier	Difficulty engaging staff and doctors to complete proper documentation	*“I feel like often it's [infections] not well documented either by the GPs* (general practitioners) *or the nursing stuff. So um it is, I often find, maybe that it did exist, the symptoms were there, they made a clinical decision under the right circumstances, but they didn't document it well, so we often fail those audits.”* (Pharmacist 5, FG5) *“it's been challenging at times to get the data, but it does show that there is a lack of documentation often, um, ah, particularly from the doctors. I'd say so um, that often I might see recorded in the progress notes, um, no symptoms.”* (Pharmacist 4, FG4) *“particularly doing the AMS or implementing AMS programs you when you're going through those just finding that an appreciation of what qualifies as a as an infection and what doesn't, what you see in the notes as opposed to what some of the online systems where you might not see anything. For instance, for a UTI It might just say, “Mavis feels unwell”, but then, when they have to report the infection that it sort of prompts them through a series of other symptoms that aren't mentioned in the notes, but you feel that they might be filling it in just so they're able to progress the report.”* (IPC Consultant 1, FG2) *“the GPs* (general practitioners) *are just very quick to not provide much detail, but just, you know the standard progress note: commence on antibiotics.”* (Director of Quality and Compliance, FG5) *“So I’m finding it very difficult, um, doing surveillance in our facilities, because the doctors write out the pathology requests, and the results go to them. So, I I have got access to like the clinical labs portal to be able to get the results, but if they don't um put, ah request the pathology on one of our pads we don't get the results. So that's the difficulty I’m having with surveillance at this present time”* (IPC Consultant 6, FG3) *“well for me, it takes a bit of following up on things because I’m not a nurse working on the floor. So it takes a lot of following up, especially if they haven't listed all symptoms things like that, or if they haven't found out what the results of the pathology … So a lot of the time we spent chasing up the results from the doctors to find out exactly what pathogen it is, etcetera.”* (IPC Lead 1, FG1)
	Barrier	Surveillance is time consuming and there is insufficient resourcing to complete it	*“Well, I've taken part in the* [AC] *NAPS, and it was time consuming, um having to go through all the, you know, documentation, trying to find doctor's notes, how they've documented looking at the infection reports, looking at pathology. Um. A lot of, I guess prep work before you could input the data.”* (IPC Lead 5, FG2) *“Well, this is my first year working with* [AC] *NAPS so I found was a little bit time consuming to put all the data, I was spending about three days, we have thirteen facilities”* (IPC Specialist 1, FG4) *“I'm panicking a bit, because some I believe it took five days and three* practitioners [to complete Aged Care NAPS]” (Pharmacist 1, FG1) *“And like everyone's saying, everyone's just so time poor”* (Pharmacist 4, FG4) *“I think the big, biggest barriers um for our staff doing that um Aged Care NAPS is staffing and availability, and the um protected time for infection control. Most of our nurses are registered nurses who have, are supposed to have some protected time, and that doesn't always happen when you have acute or unwell patients in the residential aged care facility.”* (Pharmacist 3, FG3) *“The difficulty in that is that it's still got to be resourced and there's still going to be the ability for them to have the capacity outside of their normal job to actually have time to actually do that as well. So that's the difficulty, especially with the IPCs in aged care that we’ve found, is it's fine to have some trained and sitting there and saying “that’s that, tick that box” but it’s been the resourcing of that role, and getting the ability for them to have time to actually put into that role that has been the biggest challenge.”* (IPC Consultant 2, FG2) *“the more staff the better. So, I'm not based at any site, but I must have done ten sites’* [AC] *NAPS, or at least assisted with them just because we don't have enough staff”* (IPC Consultant 5, FG3)
	Enabler	EMR improves documentation	*“We are very fortunate to have an electronic medical record and an electronic medication system. So it means that um, the infection control can, or infection surveillance can be done virtually or remotely, which makes it very easy for a lot of facilities distributed geographically, separately.”* (Pharmacist 3, FG3) *“we do have an electronic care system. I mean, we all thank God for that every day, and we have built in an infection reporting log into our system”* (Quality Manager 1, FG1) *“I think as more facilities come on to um electronic medication charts, which there's a big push over the next couple of years for that to happen, some some, but not all, of the systems, you can do Antimicrobial stewardship reports, and that will make it a lot easier, I think, for you know, on-site nurses to actually to complete the AMS surveillance”* (Pharmacist 2, FG2)
	Enabler	Shared responsibility for surveillance	*“*[surveillance]*'s part of everybody's role. It's everyone's responsibility”* [Pharmacist 1, FG1] *“we have the IPC coordinators, then we have the IPC leads, and a further down IPC support role which we have introduced at facilities, because obviously these IPC leads are, uh, they have to be the EN's or the RNs, but given the shortage of them we are, we are also encouraging our other AHPRA registered staff, such as the Allied health, and other staff members to actually uh, go through the [IPC] course and be as a support person to actually ensure that the IPC practices are done correctly, and this is actually helping us in outbreaks, because, instead of just having one person where you run the risk of them, not being able to work for many reasons. You have a team who actually goes into work. So for us we are thinking, so far it has worked”* (IPC Lead 8, FG4) *“I think it ultimately should be an IPC um job role, so they can actually um monitor the infections in their, especially in their own villages or their own sites, and then roll out some education to go with it, because you’ll find some sites have different infections to others, and sometimes you could find, you know, lead back to a cause and um monitor it from that, and do some education to the staff.”* (IPC Lead 4, FG2) *“we've done some pharmacy-based education to infection control nurses and um offered support if they have questions, or and I feel like having a point of contact with a pharmacist has been really beneficial, um, someone they can reach out to and ask questions rather than reaching out to the NAPS team.”* (Pharmacist 3, FG3)
	Enabler	Improved education will benefit all staff	*“I think that the education in aged care in general has just not been there around infection prevention… it would be useful to have some standardized guidelines that we will all agree to following”* (IPC Consultant 4, FG2) *“The Aged Care Quality Commission put out some resources late last year on urinary tract infections which I think were quite good… those kind of templates, particularly not just for nurses around surveillance, but actually the information that gets you to that point around, you know, should you be dipsticking urine… I think that would be really useful, and perhaps not just pitched at registered nurses, but perhaps pitched at you know, in that other space, particularly with the changes to aged care funding because we're going to see a shift in our in our staff and our workforce.”* (Quality Manager 1, FG1) *“having seen what we've seen with COVID and with the IPC leads, and the requirement for them to complete education, that perhaps wasn't targeted specifically to the needs of the aged care facility, that if there is going to be development of a surveillance module that it really needs to be targeted obviously to aged care.”* (IPC Consultant 3, FG2) *“I think more education around [antimicrobial stewardship] would be really beneficial, and just to you know, for me its wanting to understand it, but then to be able to like have the other RNs understand that, the other nurses, you know the Team Leaders, you know that when I’m not here, they’ll be faced with that so how do they, you know, what do they know about their knowledge and how will they manage that. So I think that would be beneficial.”* (IPC Lead 5, FG3)
Motivation	Barrier	Staff are tired and stressed from the COVID-19 pandemic	*“Everyone's getting quite stressed because their roles are just growing bigger and bigger.” (IPC Lead 4, FG2)* *“increase infections and just tiredness that goes with that. Just so, I guess, and accept an acceptance that it's here. There's infections, and it's quite hard to keep track of other things when everything is COVID related. You know it's hard to keep track of your MRSAs and your VREs when your, and you know, your life revolves around Covid.” (Quality Manager 1, FG1)* *“I think it's because aged care staff are going through so much at this time, with staff shortages, it's been a lot with the COVID situation, and I think they're just a bit worn out more than anything else, staff in aged care. That’s what I found anyway.” (IPC Lead 6, FG3)* *“with COVID and everything they're just feeling snowed under, and sometimes just let them get on with their day to day jobs is what they're asking to do. But there's more and more getting expected of them all the time.” (IPC Lead 9, FG5)*
	Barrier	Surveillance is not a priority for staff	*“in the grand scheme of things they [staff] have to do in a day, you know it's [surveillance] not at the top of their list, so there are occasions, I think, where it gets missed”* (Quality Manager 1, FG1) *“it's just not necessarily on people's priority list. Um, you know they're running around answering phones, they're picking people up off the floor, they're trying to get documentation done, following up on, and obviously talking to relatives. So um trying to add that in when it's just kind of easily dropped off their radar, I think, is problematic for us”* (Quality Manager 2, FG5) *“think sometimes the infection control just is low down on the list. Like it gets done if it gets done, and we're all happy if it gets done”* (IPC Specialist 1, FG4)
	Enabler	Utilising data for practice change	*“we have to be careful not to be collecting data for the sake of collecting data…It’s what you do with the data which is really important. And how does that data inform practice change to make improvements to care.”* (Pharmacist 4, FG4) *“We started doing NAPS, we've been doing it for three years now, and we're using that as our springboard to making continuous improvement strategies around issues that are identified in each individual home through that survey process.”* (IPC Consultant 4, FG2) *“I find that report really useful as well in education, whenever we give education to nursing staff to have data that's local to us and not just presenting it as this is happening, it's like this happens here as well as everywhere else, that can be really valuable when the pictorial graphs et cetera, can be useful for the staff to see that in a visual”* (Pharmacist 5, FG5) *“I was just going to say surveillance programs, I find they work best when when, you know, because you're collecting data, and it's what you use with the data, and I've found it quite beneficial when you actually can report back to prescribers.”* (Pharmacist 2, FG2) *“the end result was really good, um like, you know, for benchmarking purposes to see, you know where your facility is sitting, um, and yeah you can compare previous um surveys as well, so you can see how you’re tracking if the usage is increased or decreased, um, you know, looking at your trends, you know prescribing trends. Are there patterns picking up on areas that “okay, this is happening repeatedly, um, you know, right, what can I do about it?” So it gives you a good idea, about your position, and you know where you're sitting.”* (IPC Lead 5, FG3) *“every year we do participate in the NAPS and then we run an organization-wide report. From there onwards we actually do run improvement projects at our facility, at a facility like for this year we did identify a bit of an increase in the skin infections and then we combine that NAPS surveillance data with our internal monitoring analysis and then we actually found that “ah!” there was actually a correlation between COVID outbreaks and skin infection. So, at the moment we are actually on the phase one of our Improvement project so hopefully by the end of the year we will see a reduction in that”* (IPC Lead 8, FG4)

### Infection surveillance not widely understood by RACF staff (Capability—Barrier)

Participants reported that RACF healthcare staff who do not have direct involvement in entering surveillance data often lack an awareness of what surveillance is and how to apply guidelines and enter appropriate data.

This was supported by the survey, with respondents indicating that *a lack of expertise in surveillance* and *limited skilled personnel* were common barriers to participation in surveillance programs (Table 2).

### Previous experience with infection surveillance improves understanding (Capability—Enabler)

Participants in the focus groups were all involved directly in infection surveillance. All had an understanding of infection surveillance principles and felt comfortable with their knowledge.

Previous participation in infection surveillance, including Aged Care NAPS, improved understanding of surveillance and ability to identify and report infections.

### Difficulty engaging staff and doctors to complete proper documentation (Opportunity—Barrier)

Participants reported that RACF staff and visiting doctors often do not provide enough detail in their documentation of infections. In particular, it was reported that doctors fail to engage in elements of antimicrobial stewardship, providing only minimal detail of why antibiotics have been commenced. This resulted in concerns that surveillance *“doesn't get reported as well as it potentially could”* leading to *“adequate surveillance”* rather than *“best practice surveillance”* (Quality Manager 1, FG1). A third of survey respondents reported having more than 6 primary care physicians visit their facilities (*n* = 60 34.9%).

While a small number of participants reported the need to follow up with primary care physicians about pathology results, the survey showed that most participants have direct access to pathology results for individual residents (*n* = 141, 86%) and/or a simplified summary of results (*n* = 118, 72.4%) for those residents who had pathology specimens taken. Access to data and pathology results for these residents were not commonly identified barriers (Table 2).

### Surveillance is time consuming and there is insufficient resourcing to complete it (Opportunity—Barrier)

RACF nursing and IPC staff have large workloads and are *“inundated … with just providing the most basic of care”* (IPC Consultant 3, FG2), which impacts on their ability to participate in surveillance. Participants felt that surveillance is time consuming and that they have little time to dedicate to it. Similarly, survey participants indicated that the considerable time commitment required for surveillance was the most common barrier to participation (Table 2).

These high workloads were compounded by insufficient resources to fill positions and complete tasks, with one participant noting that *“staffing is just an ongoing daily issue for all of our aged care facilities”* (IPC Consultant 7, FG4).

### Electronic medical records improve documentation (Opportunity—Enabler)

Most survey participants (82.8%) stated that their facility currently uses electronic medical records (EMR). Most participants reported that their EMR programs capture details of infections (*n* = 119, 93%), vaccinations (*n* = 115, 89.8%), medications (including antimicrobials) (*n* = 100, 78.1%) and pathology results (*n* = 95, 74.2%). Focus group participants discussed how EMR has improved entry of surveillance data, with paper charts being *“time consuming”* (Pharmacist 1, FG1) and an *“absolute nightmare”* (IPC Consultant 4, FG2), whereas EMR have streamlined and simplified processes.

### Shared responsibility for surveillance (Opportunity—Enabler)

IPC leads and consultants were most frequently responsible for entering surveillance data. However, surveillance was seen as a shared responsibility for healthcare staff in RACFs, with some participants highlighting the importance of all staff understanding surveillance methodology to allow for redundancy if specialist staff are unavailable. Equally, staff with specialist knowledge and training were also seen as vital for guiding surveillance and helping to train staff and implement correct data collection. Almost all RACFs have access to an IPC lead, with only 6.2% (*n* = 11) reporting they currently have none in their facilities. In addition, 44.9% (*n* = 75) of survey respondents reported having an IPC coordinator available through their provider group or health service.

### Improved education will benefit all staff (Opportunity—Enabler)

Participants felt that the current education about surveillance and antimicrobial stewardship was not sufficient and it emerged that improved education would increase participation in surveillance activities.

Participants felt it was important to have resources specific to aged care and tailored information for all levels of RACF staff to allow them to be more involved in surveillance. Topics nominated by participants included infection prevention, infection surveillance in aged care, and case studies. Surveyed respondents reported a preference for education regarding surveillance methodology, interpretation of surveillance reports and principles of antimicrobial use. Most respondents preferred on-line modalities of education, with webinars, on-line resources and self-guided on-line training being the most popular.

### Staff are tired and stressed from the COVID-19 pandemic (Motivation—Barrier)

Participants expressed that they and their colleagues were tired and stressed, particularly after managing the COVID-19 pandemic in their facilities. They felt that *“COVID’s pretty much taken over our world in a lot of ways”* (NUM, FG4) but there are still high expectations of what staff in RACFs must undertake on a daily basis. One called for an *“understanding of what is reasonable and what isn't”* (Quality Manager 1, FG1) for RACF staff.

### Surveillance is not a priority for staff (Motivation—Barrier)

Participants revealed that provision of clinical care is the highest priority for them and other RACF staff, and that they often have a number of other (non-surveillance) time-sensitive tasks to complete. Frequently, surveillance activities are pushed lower on their task list each day. This was supported by survey results, which identified competing priority tasks as the second most common barrier to participation in surveillance (Table 2).

### Utilising data for practice change (Motivation—Enabler)

Participants revealed that it was important for there to be a purpose to collecting surveillance data. They explained that this was to ensure that the time commitment and effort required from staff to complete it was regarded as worthwhile.

Results from Aged Care NAPS and internal audits are commonly used by RACF staff to bring about practice change and improvements, to compare performance against previous years, and to train staff. The site-specific data from surveillance provides unique evidence to support initiation of these activities by IPC staff. Participants appreciated the importance of participating in surveillance when there was a practical, known use for the data.

Similarly, survey findings revealed that staff use surveillance data, with 75.4% of respondents saying that infection and antimicrobial use reports are fed back to a multidisciplinary committee for review, and 74.5% of these respondents saying the committee finds it helpful if the reports enable benchmarking.

## Discussion

This study aimed to identify the barriers and enablers to participation in infection surveillance in Australian RACFs, to improve development and implementation of a national, standardised infection surveillance program. This is the first time that an in-depth qualitative analysis of the two currently available surveillance programs has been conducted from a staff perspective.

Audit and feedback of data is well-recognised as a motivator for behaviour change in the healthcare sector [27, 28]. Specifically, infection surveillance allows RACFs to audit their progress against previous performance and benchmark with other facilities to encourage participation in surveillance [8, 9]. Participants in our study noted the benefits of receiving surveillance reports for initiating practice change and educating staff. Creating change and having a positive outcome from program implementation can improve confidence of staff and encourage participation despite other barriers [29]. It is important that all RACF staff understand the benefit of collecting surveillance data to ensure it is collected with purpose. Implementation of NISPAC would be strengthened by clear, easy to understand reports and education for staff regarding the interpretation and use of data to improve local practices.

Participants in the survey and focus groups felt that knowledge among RACF staff and education about infection surveillance and related topics was lacking. Previous studies engaging IPC staff, including in Australian RACFs, have similarly found an absence of access to IPC education, with one study reporting no education programs about infection surveillance provided in surveyed facilities [30–32]. Consistent with prior studies, our study found that IPC staff are most often responsible for infection surveillance activities in RACFs [30]. IPC staff are now required by the Australian Government to complete specific IPC training [20]. However, focus group participants highlighted that there is a need to educate other RACF staff to complete surveillance so that the responsibility can be shared, and ensure that surveillance processes can be completed in the absence of IPC staff. Workforce shortages are an ongoing issue in Australian RACFs and this was compounded during the height of the COVID-19 pandemic, which highlighted a lack of staff skilled in infection control [33, 34]. Implementation of a new surveillance program with education modules that are applicable to all levels of RACF healthcare staff may help to ease workloads by creating shared responsibility.

The COVID-19 pandemic greatly impacted RACFs in Australia, particularly in the state of Victoria where a majority of the focus group participants were located [35]. The COVID-19 pandemic was raised by participants as a barrier to conducting surveillance, due to increased stress and high expectations placed on staff. Staff in RACFs experienced work-related stress, exhaustion and higher workloads due to the pandemic [36]. COVID-19 outbreaks are still regularly occurring at Australian RACFs, and it is likely that the added workload from these will continue to impact RACF staff into the foreseeable future [37].

Similarly, staff reported the time needed to complete surveillance and other clinical priorities as barriers to surveillance activities. Time constraints, competing priority tasks and workloads have been identified as barriers to participating in infection surveillance programs internationally, and in other quality improvement initiatives in Australia and overseas prior to the pandemic [9, 32, 38–41]. These are important considerations for development of NISPAC. Implementation of NISPAC must streamline surveillance activities into usual workflows and keep the time required by staff to participate to a minimum. Similarly, surveillance data must be consistent with data already captured via EMR systems, therefore a synergy between EMR and surveillance may need to be explored to reduce duplicity and increase efficiency.

A lack of engagement from clinicians can hinder implementation of quality improvement programs in the aged care setting [38, 42]. Poor understanding from clinicians of the importance of detailed documentation led to difficulty for staff in successfully entering surveillance data in this study. Engagement from clinicians may improve uptake of surveillance in aged care through demonstrating to RACF staff the benefit of participation and by improving quality of patient data. Some facilities reported having many individual primary care physicians visiting, which may make engagement difficult. Previous studies have also noted difficulties in engaging offsite primary care physicians in IPC activities and reviewing antimicrobials [32, 43]. Future studies may be needed to understand physician attitudes to surveillance in RACFs, and to understand how best to report surveillance findings to this stakeholder group and support their engagement with surveillance.

Potential study limitations include the fact that although an online medium was used to provide a convenient tool for focus group participants across Australia, the platform was not always conducive to fluency and continual dialogue by participants. Prompting by the facilitator was necessary to support discussion. The consistency on most topics between the survey and focus groups results indicates that the impact of this was minimal. Further, the studied cohorts may represent a select subset of staff, rather than being truly representative of all Australian facilities. However, we note individuals from multiple jurisdictions across Australia participated, and a range of different experience levels were noted across the survey and focus groups.

## Conclusion

This study identified several barriers and enablers to participation in two infection and antimicrobial use surveillance programs in Australian RACFs. It is vital that the perspectives of staff are considered in the development and implementation of future surveillance initiatives, particularly in light of significant impacts of the COVID-19 pandemic upon workforce and workload of staff. This study has identified that workloads and pressure on staff must be considered, and that staff are eager for tailored education to improve participation in a standardised surveillance network. Surveillance reporting and feedback to staff is also important and can provide a basis for quality improvement initiatives. The understanding of staff perspectives gained from this study will be incorporated into implementation materials and strategies underpinning NISPAC.

### Supplementary Information

**Additional file 1. **Focus group guide.

**Additional file 2. **Survey results.

## Data Availability

The datasets generated and analysed during the current study are not publicly available to maintain privacy of individuals but are available from the corresponding author on reasonable request.

## Abbreviations

ACIIP: Aged Care Infection Indicator Program
Aged Care NAPS: Aged Care National Antimicrobial Prescribing Survey
COVID-19: SARS CoV-2 virus
IPC: Infection Prevention and Control
NCAS: National Centre for Antimicrobial Stewardship
NISPAC: National Infection Surveillance Program for Aged Care
RACF: Residential Aged Care Facility
VICNISS: The Victorian Healthcare Associated Infection Surveillance System
