# Supplementary material for: Identifying barriers and enablers to participation in infection surveillance in Australian residential aged care facilities
Source: BMC Public Health. 2023 Nov 4;23:2160. doi: 10.1186/s12889-023-16891-2 (PMC10625226; doi:10.1186/s12889-023-16891-2)
Supplement: Supplementary file 1 — Additional file 1. Focus group guide. [file 12889_2023_16891_MOESM1_ESM.docx]

**Additional file 1 - Focus group guide**

| **Question** | **COM-B domain** | **Notes** |
| --- | --- | --- |
| Q1. What is your understanding of infection surveillance? | Capability |  |
| Q2. Tell me about your previous experience with infection surveillance programs in aged care. | Capability |  |
| Q3. Can you tell me who was responsible for infection surveillance in your facility? Who do you think should be responsible? | Opportunity |  |
| Q4. What have been some of the challenges of participating in infection surveillance programs at your facility? What do you think would be some of the challenges with implementing a new program at your facility? *Prompts: priorities, time commitment, resources, staff* | Motivation, Opportunity |  |
| Q5. What do you think would help you to participate in infection surveillance in your facility? *Prompts: Education + education types, resources, managerial support* | Motivation, Opportunity |  |
| Q6. What do you think would help your facility to introduce an infection surveillance program? *Prompts: Staffing, training, benchmarking, reports* | Opportunity |  |
| Q7. Any other comments you wish to make? |  |  |
