# Supplementary material for: Identifying barriers and enablers to participation in infection surveillance in Australian residential aged care facilities
Source: BMC Public Health. 2023 Nov 4;23:2160. doi: 10.1186/s12889-023-16891-2 (PMC10625226; doi:10.1186/s12889-023-16891-2)
Supplement: Supplementary file 2 — Additional file 2. Survey results. [file 12889_2023_16891_MOESM2_ESM.docx]

**Additional file 2 - Survey results**

*Human Resources – Infection Prevention and Control*

| **Question** | **n(%)** |
| --- | --- |
| How many Aged Care IPC Leads, as specified by the Australian Government Department of Health are currently employed in your RACF?  Currently none  1  2  3 or more | 11 (6.2)  92 (52)  41 (23.2)  33 (18.6) |
| In addition to the Aged Care IPC Leads, are IPC consultants employed at your RACF?  Yes, an IPC coordinator is appointed for our RACF provider group or health service  Yes, external IPC consultants are permanently employed  Yes, external IPC consultants are periodically employed  Yes, other  No | 75 (44.9)  9 (5.4)  13 (7.8)  7 (4.2)  63 (37.7) |
| How many General Practitioners visit your RACF?  1-5  6-10  11 or more | 112 (65.1)  37 (21.5)  23 (13.37) |

*Pathology*

| **Question** | **n(%)** |
| --- | --- |
| Do onsite RACF clinical staff have access to pathology results?  Yes  No | 141 (86)  23 (14) |
| Are the pathology results electronically available (versus faxed or mailed)?  Yes  No | 122 (86.5)  19 (13.5) |
| Do IPC staff have access to summarised pathology results for all residents?  Yes  No | 118 (72.4)  45 (27.6) |

*Information Technology*

| **Question** | **n(%)** |
| --- | --- |
| Does your RACF use electronic records for each resident?  Yes  No | 130 (82.8)  27 (17.2) |
| Do these electronic records include information about:  Resident demographics  Infections  Pathology results  Medications, including antimicrobials  Resident vaccinations  other | 124 (96.9)  119 (93)  95 (74.2)  100 (78.1)  115 (89.8)  15 (2.6) |

*Surveillance*

| **Question** | **n(%)** |
| --- | --- |
| Are both infection and antimicrobial use surveillance reports fed back to a multidisciplinary committee for review?  Yes  No  Unsure | 98 (75.4)  16 (12.3)  16 (12.3) |
| Does the committee find it helpful if the surveillance reports enable benchmarking of their RACF rates (performance) against an external pooled rate?  Yes  No  Not possible, data is for internal use only  Unsure | 73 (74.5)  3 (3.1)  13 (13.8)  9 (9.2) |

*Education*

| **Question** | **n(%)** |
| --- | --- |
| To assist with participation in surveillance programs, which of the following educational sessions might the Aged Care IPC Lead(s) find helpful?  Significant organism infections (e.g. CPE)  Principles of antimicrobial use  Employee health and immunisation  Surveillance methodology  Interpretation of surveillance reports  Practical IT guidance on how to use web forms  Other | 90 (73.2)  95 (77.2)  65 (52.9)  98 (79.7)  94 (76.4)  54 (43.9)  5 (4.1) |
| Which mode of education would the Aged Care IPC Lead(s) prefer?  Face-to-face workshops/seminars  Onsite interactive training sessions (e.g. hand hygiene)  Webinar  Online resources (e.g. user guides)  Self-guided online training sessions  Phone  Other | 78 (63.9)  71 (58.2)  88 (72.1)  83 (68)  83 (68)  13 (10.7)  2 (1.6) |
